# Retraction Note: Combined chemo-magnetic field-photothermal breast cancer therapy based on porous magnetite nanospheres

**DOI:** 10.1038/s41598-026-70446-0

**Published:** 2026-09-14

**Authors:** Majid Sharifi, Anwarul Hasan, Nadir Mustafa Qadir Nanakali, Abbas Salihi, Fikry Ali Qadir, Hawzheen A. Muhammad, Mudhir Sabir Shekha, Falah Mohammad Aziz, Karwan M. Amen, Farrokh Najafi, Hasan Yousefi-Manesh, Mojtaba Falahati

**Affiliations:** 1 Department of Nanotechnology, Faculty of Advanced Sciences and Technology, Tehran Medical Sciences, Islamic Azad University, Tehran, Iran; 2 Department of Animal Science, Faculty of Agriculture, University of Tabriz, Tabriz, Iran; 3 Biomedical Research Center, Qatar University, 2713 Doha, Qatar; 4 Department of Mechanical and Industrial Engineering, College of Engineering, Qatar University, 2713 Doha, Qatar; 5 Department of Biology, College of Science, Cihan University-Erbil, Kurdistan Region, Iraq; 6 Department of Biology, College of Education, Salahaddin University-Erbil, Kurdistan Region, Iraq; 7 Department of Biology, College of Science, Salahaddin University-Erbil, Kurdistan Region, Iraq; 8 Department of Microbiology, College of Medicine, University of Sulaimani, Sulaimani, Kurdistan Region Iraq; 9 Department of Medical Analysis, Faculty of Science, Tishk International University, Erbil, Iraq; 10 College of Nursing, Hawler Medical University, Erbil, Iraq; 11 Department of Biomaterial Engineering, University of Amir-Kabir, Tehran, Iran; 12 Department of Pharmacology, School of Medicine, Tehran University of Medical Sciences, Tehran, Iran

Retraction of: *Scientific Reports* https://doi.org/10.1038/s41598-020-62429-6, published online 03 April 2020

The Editors have retracted this Article.

After publication, concerns were raised regarding the two-dimensional contour density plots of 4T1 cells in Figure 4A. Specifically, the plots 2 and 5 appear highly similar to each other. The Authors were unable to provide the original raw data to address the concerns. The Editors therefore no longer have confidence in the reliability of the results and conclusions presented.

Authors did not respond to correspondence from the Editors about this retraction.

